# Global research architecture of RNA-binding proteins in neurodegenerative diseases: a web of science bibliometric study with PubMed record verification, 2001–2025

**DOI:** 10.3389/fnagi.2026.1967205

**Published:** 2026-09-21

**Authors:** Ranran Zhu, Hongyan Li

**Affiliations:** 1 Graduate School, Xinjiang Medical University, Urumqi, Xinjiang, China; 2 Department of Neurology, People’s Hospital of Xinjiang Uygur Autonomous Region, Urumqi, Xinjiang, China

**Keywords:** bibliometric analysis, cryptic exons, frontotemporal dementia, liquid–liquid phase separation, neurodegeneration, RNA splicing, RNA-binding proteins, TDP-43

## Abstract

**Background:**

RNA-binding proteins (RBPs) connect RNA homeostasis to proteostasis and selective neuronal vulnerability, but the organization and evolution of this cross-disease research field remain unclear.

**Methods:**

We analyzed Web of Science Core Collection (WoSCC) records published through 2025. PubMed was used only to verify records by normalized DOI or exact normalized title; PubMed metadata were not pooled with WoS data, and all bibliometric analyses used WoS fields. Publication growth, contributors, collaboration, co-citation, bibliographic coupling, and conceptual evolution were examined using bibliometrix, VOSviewer, and CiteSpace-compatible workflows. A prespecified 43-concept dictionary preserved multiword biological entities in title/abstract networks. The study characterizes a query-defined and PubMed-verified literature corpus rather than estimating the relative biological importance of individual RBPs.

**Results:**

Of 2,454 records reported by the WoS search interface, 2,450 were available in the supplied exports and 2,309 met the year criterion. PubMed record verification retained 2,160 records (93.55%) in the analysis corpus; 149 records (6.45%) were not matched. Output increased from one publication in 2001 to 213 in 2025. Within this query-defined corpus, the mapped literature was concentrated around TDP-43 pathology, TARDBP/FUS genetics, and loss of RNA-processing function. The controlled concept network resolved three macro-communities, while recent temporal signals involved LATE, cryptic exons, and RNA transport. These findings describe the retrieved and verified corpus and are not presented as a search-independent ranking of the broader RBP field.

**Conclusion:**

The maps document a WoS-derived and PubMed-verified literature corpus concentrated on TDP-43 and ALS/FTD and increasingly connected with cryptic RNA processing, condensate biology, nuclear transport, neuroimmune responses, and aging-related mixed proteinopathies. Because the corpus was conditioned by a title-level query containing named RBPs and by PubMed record verification, these patterns should not be interpreted as an unbiased ranking of RBP biology or as evidence of shared causality across diseases. Cross-disease translation should proceed through neuropathologically defined, age-stratified, and cell-type-resolved human cohorts, followed by causal perturbation and rescue in human disease models and multicenter longitudinal validation.

## 1 Introduction

RNA-binding proteins coordinate pre-mRNA processing, alternative splicing, polyadenylation, nuclear export, subcellular RNA localization, stability, and translation. These functions are particularly consequential in neurons, whose longevity, polarized morphology, and dependence on local translation create sustained demands on RNA quality control (Nussbacher et al., 2015; Geuens et al., 2016). RBP dysfunction can therefore combine loss of nuclear RNA regulation with cytoplasmic mislocalization, altered ribonucleoprotein granules, impaired axonal transport, and protein aggregation. This convergence has become a major framework for understanding amyotrophic lateral sclerosis (ALS), frontotemporal dementia (FTD), and related neurodegenerative disorders (Taylor et al., 2016).

The identification of TAR DNA-binding protein 43 (TDP-43) as the principal ubiquitinated protein in most ALS and frontotemporal lobar degeneration (FTLD) inclusions reorganized the field (Neumann et al., 2006; Arai et al., 2006). Pathogenic variants in *TARDBP* and *FUS*, together with the biochemical definition of phosphorylated TDP-43 pathology, established a direct connection between RBPs and neurodegeneration (Sreedharan et al., 2008; Vance et al., 2009; Hasegawa et al., 2008). Transcriptome-wide studies then showed that nuclear TDP-43 loss perturbs long neuronal transcripts and position-dependent splicing (Polymenidou et al., 2011; Tollervey et al., 2011), while subsequent work identified repression of cryptic exons as a central loss-of-function mechanism (Ling et al., 2015). In parallel, low-complexity domains, stress granules, and liquid-to-solid transitions provided a physical framework linking physiological RBP assembly to persistent pathological states (Molliex et al., 2015; Patel et al., 2015; Wolozin and Ivanov, 2019). The recognition of limbic-predominant age-related TDP-43 encephalopathy (LATE) further extended TDP-43 biology from ALS/FTD into common late-life cognitive impairment and mixed neuropathology (Nelson et al., 2019).

This literature now spans neuropathology, human genetics, transcriptomics, biophysics, cell biology, immunology, and translational neuroscience. Narrative reviews can synthesize mechanisms, but they do not quantify the field’s path dependence, collaboration structure, knowledge base, or relative integration of diseases and mechanisms. Bibliometric science mapping offers complementary tools for evaluating these features (Aria and Cuccurullo, 2017; van Eck and Waltman, 2010; Chen, 2006; Donthu et al., 2021). However, biomedical bibliometric studies are vulnerable to database-specific coverage, author and institution ambiguity, citation-age bias, and semantic artifacts produced when multiword entities are split into isolated tokens.

We therefore conducted a bibliometric study of RBP research across neurodegenerative diseases. WoSCC was the sole analytical database and supplied the citation, cited-reference, keyword, and affiliation fields used for science mapping; PubMed served only as an external source for record-level verification. Unlike prior bibliometric studies centered on ALS, FTD, or a small set of highly cited neurodegeneration papers (Shi et al., 2022; Chen et al., 2024; Liao et al., 2021), our unit of analysis was RBP research spanning multiple neurodegenerative diseases. We aimed to: (i) characterize publication growth and contributors; (ii) define collaboration and co-citation structures; (iii) map conceptual architecture without fragmenting protected biological phrases; (iv) identify temporal shifts; and (v) assess how the dominant ALS/FTD program connects with aging-related and other disease literatures. The record-selection diagram was adapted from PRISMA 2020 for transparency, without classifying this bibliometric study as a systematic review (Page et al., 2021).

## 2 Materials and methods

### 2.1 Study design and data sources

This original bibliometric study used Web of Science Core Collection (WoSCC) as the sole analytical database because its cited-reference and affiliation fields support reproducible science mapping. PubMed served only as an external record-level verification source during primary-corpus selection. PubMed citation counts, keywords, references, affiliations, and other analytical fields were neither merged with nor substituted for WoS fields.

The WoS search was conducted on 21 June 2026. Eligible publication years were 2000–2025, allowing a complete calendar-year endpoint. The stored query was:

TI = (“RNA-binding protein*” OR “RNA binding protein*” OR “RBP” OR “TDP-43” OR “FUS protein” OR “hnRNP*” OR “FMRP” OR “Musashi” OR “YTHDF*” OR “G3BP*” OR “ELAVL*” OR “HuD”) AND TS = (“neurodegenerative disease*” OR “neurodegeneration” OR “Alzheimer*” OR “Parkinson*” OR “amyotrophic lateral sclerosis” OR “ALS” OR “frontotemporal dementia” OR “FTD” OR “Huntington*”).

The RBP component was restricted to the title to improve topic specificity, whereas disease terms could occur in the topic field. The search was designed to characterize the research architecture captured by this prespecified title-anchored query, not to estimate the relative biological importance or literature-wide prevalence of individual RBPs. English-language records whose primary WoS document type was Article or Review were eligible. Secondary labels such as Early Access, Proceedings Paper, or Book Chapter were retained when the primary type remained Article or Review. Full records and cited references were exported.

### 2.2 Record verification and selection

The WoS interface reported 2,454 results. Five supplied text exports contained 2,450 parseable records, leaving four interface records unavailable for evaluation. Records were restricted to the prespecified publication years before matching. DOI values were lowercased and stripped of URL and prefix artifacts; titles underwent Unicode NFKD normalization, lowercasing, and removal of non-alphanumeric characters. A WoS record entered the primary corpus when either its normalized DOI or exact normalized title matched a PubMed record. DOI and title both matched for 2,129 records, DOI alone for 18, and title alone for 13. A failure to match was treated as a database-verification outcome, not as an assessment of study quality. PubMed metadata were not used in the bibliometric analyses. Duplicate checks were repeated after matching.

### 2.3 Data cleaning and semantic quality control

The WoS accession number was the primary record identifier. Raw author keywords and Keywords Plus were preserved as DE_RAW and ID_RAW. A conservative thesaurus harmonized unambiguous variants, including ALS/amyotrophic lateral sclerosis, Alzheimer disease/Alzheimer’s disease, FTD/frontotemporal dementia, FTLD/frontotemporal lobar degeneration, stress granule(s), RBP/RNA-binding protein(s), and LLPS/liquid–liquid phase separation. FTD and FTLD were retained separately because they represent related but non-identical clinical and pathological constructs.

Title and abstract concept analyses used a prespecified dictionary of 43 disease, RBP, cellular-process, and pathological phrases. Whole-phrase and explicit-acronym matching occurred before network construction. Thus, “amyotrophic lateral sclerosis” remained one node and did not also produce redundant amyotrophic, lateral, or sclerosis nodes. Generic terms such as protein, disease, expression, and gene were excluded from the curated combined-keyword sensitivity network. Every cleaning rule and applied replacement was logged.

### 2.4 Descriptive and citation analyses

Annual and cumulative publication counts, total citations, and mean citations were calculated by publication year. A second-order polynomial model was used only as a descriptive summary of observed growth; it was not interpreted as a causal model or forecast. Citation rankings were reported as both total citations and citations per citable year to reduce, but not eliminate, citation-age bias. Bradford’s law was used to identify the journal core.

Countries were extracted from all available affiliation addresses and counted once per document per country using full counting. A document with affiliations in one country was classified as a single-country publication (SCP); each represented country received a multiple-country publication (MCP) count when two or more countries appeared. Country totals are therefore not mutually exclusive across countries. Organizations were derived from the WoS C3 standardized field; umbrella systems and constituent campuses were not summed.

### 2.5 Network and temporal analyses

VOSviewer workflows were used for co-authorship, co-citation, bibliographic-coupling, and co-occurrence networks. Association-strength normalization and VOS clustering were applied. The main country map retained 40 connected countries, the main co-cited-reference map retained 60 core references, and the primary conceptual analysis used all 43 controlled title/abstract phrases. Node size denotes occurrence or weight, link thickness denotes relationship strength, cluster color denotes community membership, overlay color denotes mean publication year, and the density view represents the weighted concentration of neighboring concepts.

CiteSpace-compatible analyses used one-year slices across 2001–2025. The complementary reference network applied g-index selection with *k* = 25 and no pruning. For keyword analyses, controlled phrases were written as semicolon-delimited inline fields to prevent continuation-line concatenation. Kleinberg burst detection used gamma = 0.30 and a minimum duration of two years. The 25 highest-strength bursts were displayed from 32 detected items. Network specifications and the distinct roles of the main and sensitivity analyses are summarized in Table 1.

**Table 1:** Specifications and interpretive roles of the principal network analyses.

Analysis	Software/view	Unit	Selection	Nodes	Links	Normalization or clustering	Role in interpretation
Country collaboration	VOSviewer	Countries	Top 40 connected countries	40	264	Association strength and VOS clustering	Main social-structure map
Reference co-citation	VOSviewer	Cited references	60 core references	60	1770	Association strength and VOS clustering	Main knowledge-base map
Controlled concept co-occurrence	VOSviewer	Title/abstract phrases	43 prespecified phrases	43	682	Association strength and VOS clustering	Main conceptual map
Controlled concept overlay	VOSviewer	Title/abstract phrases	Same 43 phrases	43	682	Mean publication year	Temporal interpretation
Controlled concept density	VOSviewer	Title/abstract phrases	Same 43 phrases	43	682	Weighted item density	Concept concentration
Reference sensitivity network	CiteSpace-compatible	Cited references	g-index k = 25 by annual slice	80	221	No pruning; Q = 0.4679; S = 1.000	Sensitivity analysis
Controlled keyword network	CiteSpace-compatible	Controlled phrases	43 phrases with inline semicolon parsing	43	120	CiteSpace-compatible clustering	Supplementary structural view
Controlled keyword timeline	CiteSpace-compatible	Controlled phrases	Same 4 3 phrases	43	536	3 clusters; Q = 0.0663; S = 0.7442	Supplementary temporal view
Controlled keyword bursts	CiteSpace-compatible	Controlled phrases	Kleinberg gamma = 0.30; minimum 2 years	32 detected	25 displayed	Burst strength and interval	Supplementary emerging-front analysis

Link definitions and selection rules differ across software and views; link counts should not be compared as if they measured the same quantity. *Q*, modularity; *S*, weighted mean silhouette.

### 2.6 Reproducibility and within-corpus triangulation

Conceptual findings were triangulated across author keywords, Keywords Plus, controlled title/abstract phrases, VOSviewer overlays, and CiteSpace-compatible bursts. This within-corpus triangulation assessed whether related patterns recurred across analytical fields and methods; it was not treated as evidence that the findings were insensitive to database selection or query formulation. Outputs were accepted only after semantic inspection; a dual-map output with zero matched journal pairs and earlier uncurated keyword outputs were excluded from interpretation. Missingness, database discrepancies, counting rules, thesaurus changes, software versions, thresholds, and rejected outputs were retained in an execution audit.

Analyses used R 4.2.3, bibliometrix 5.2.1, VOSviewer 1.6.20 workflows, CiteSpace-compatible DS-Agent Analysis modules, and Python 3.11. Project-specific scripts, derived tables, parameter files, and logs are retained. OpenAI Codex (GPT-5, OpenAI, https://openai.com) assisted with language editing, code review, and preparation of reproducible visualization workflows; all data, code, references, figures, and interpretations were checked by the authors.

## 3 Results

### 3.1 Record selection and corpus characteristics

After removal of 141 records outside the eligible years or with invalid year data, 2,309 WoS records remained. PubMed verification excluded 149 records for which neither the normalized DOI nor the exact normalized title matched, yielding 2,160 included publications and no post-match duplicates (Figure 1). The observed series began in 2001 because no verified eligible record was published in 2000.

**Figure 1:**
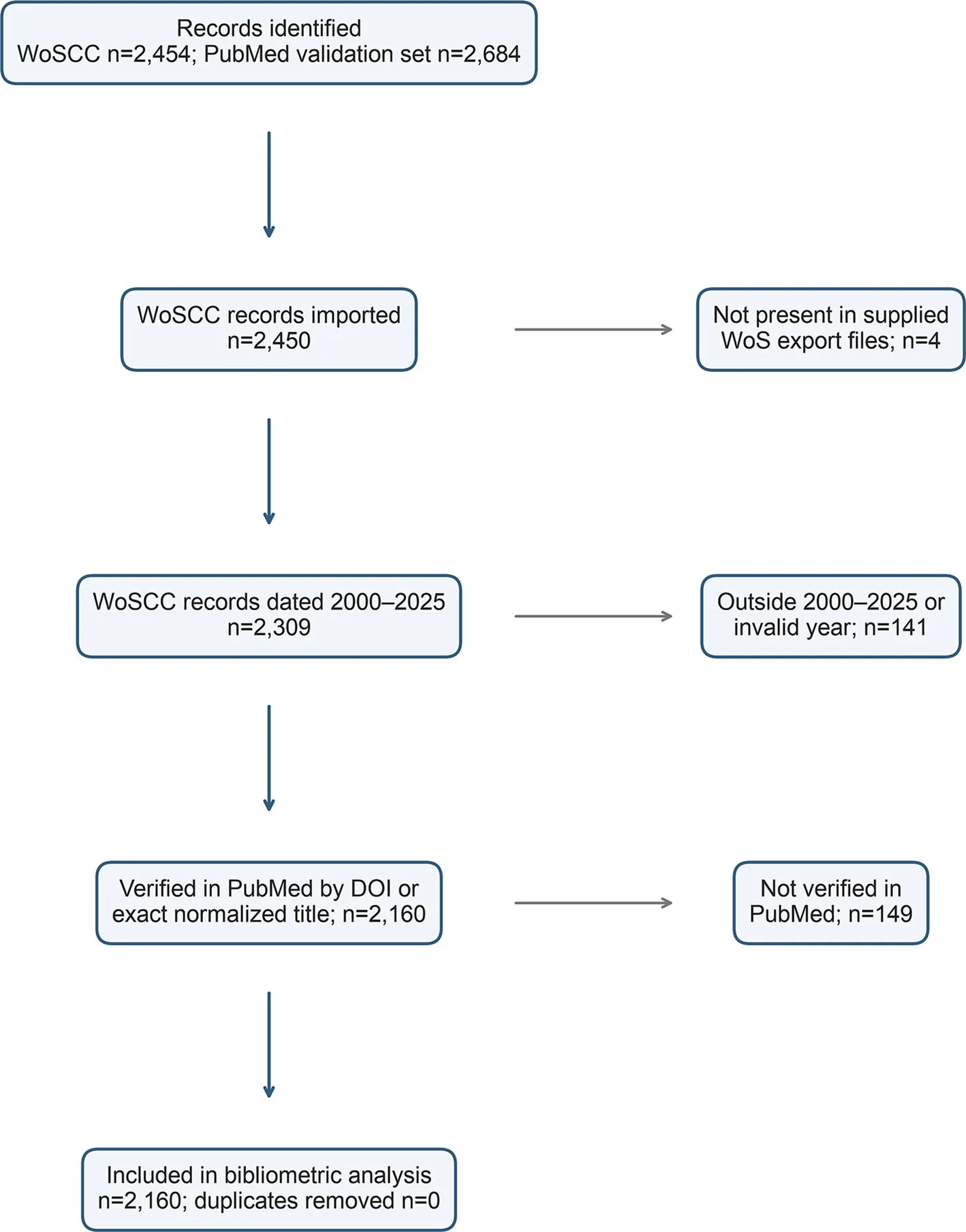
Study identification and PubMed record verification. WoSCC was the sole bibliometric data source. PubMed was used only to verify eligible WoS records by normalized DOI or exact normalized title; PubMed analytical fields were not pooled with WoS data. Adapted from the PRISMA 2020 flow diagram (Page et al., 2021), licensed under CC BY 4.0.

The corpus contained 1,895 Articles, 246 Reviews, and 19 records with an eligible Article/Review primary type plus another retained subtype. It spanned 404 sources, 10,520 authors, 1,405 standardized organizations, and 53,381 distinct cited-reference strings. The publications had accumulated 143,144 citations (mean 66.27 per document). Twelve records lacked abstracts, 717 lacked author keywords, 39 lacked Keywords Plus, four lacked affiliation addresses, and eight lacked DOI values. One record carried a Retracted Publication flag and was retained in the fixed analysis set but not used as mechanistic evidence (Table 2).

**Table 2:** Corpus characteristics, selection, and field completeness.

Domain	Measure	Value
Selection	WoSCC results reported by interface	2,454
Selection	Records available in supplied exports	2,450
Selection	Records after year validation	2,309
Selection	WoS records verified in PubMed and included	2,160
Coverage	Observed publication years	2001–2025
Corpus	Articles	1895
Corpus	Reviews	246
Corpus	Other retained article/review subtypes	19
Corpus	Sources	404
Corpus	Authors	10,520
Corpus	Standardized organizations	1,405
Impact	Total citations	143,144
Impact	Mean citations per document	66.27
Missingness	Missing abstracts	12 (0.56%)
Missingness	Missing author keywords	717 (33.19%)
Missingness	Missing Keywords Plus	39 (1.81%)
Missingness	Missing affiliation addresses	4 (0.19%)
Missingness	Missing DOI	8 (0.37%)
Integrity	Retracted-publication flags retained	1

Percentages for missingness use the 2,160-record analysis set as the denominator. The 19 other retained subtypes had Article or Review as their primary WoS document type.

### 3.2 Growth and publication venues

Annual output remained below 20 publications through 2007, rose to 71 in 2010, exceeded 100 in 2017, and reached 155 in 2020. Output increased to 198 in 2021, fluctuated between 167 and 175 during 2022–2024, and reached a new maximum of 213 in 2025. The quadratic descriptive model explained 95.99% of observed annual variation (Figure 2). Citation totals were concentrated in older discovery cohorts; the two included 2006 papers accumulated 7,597 citations, demonstrating why output and impact trajectories should be interpreted separately.

**Figure 2:**
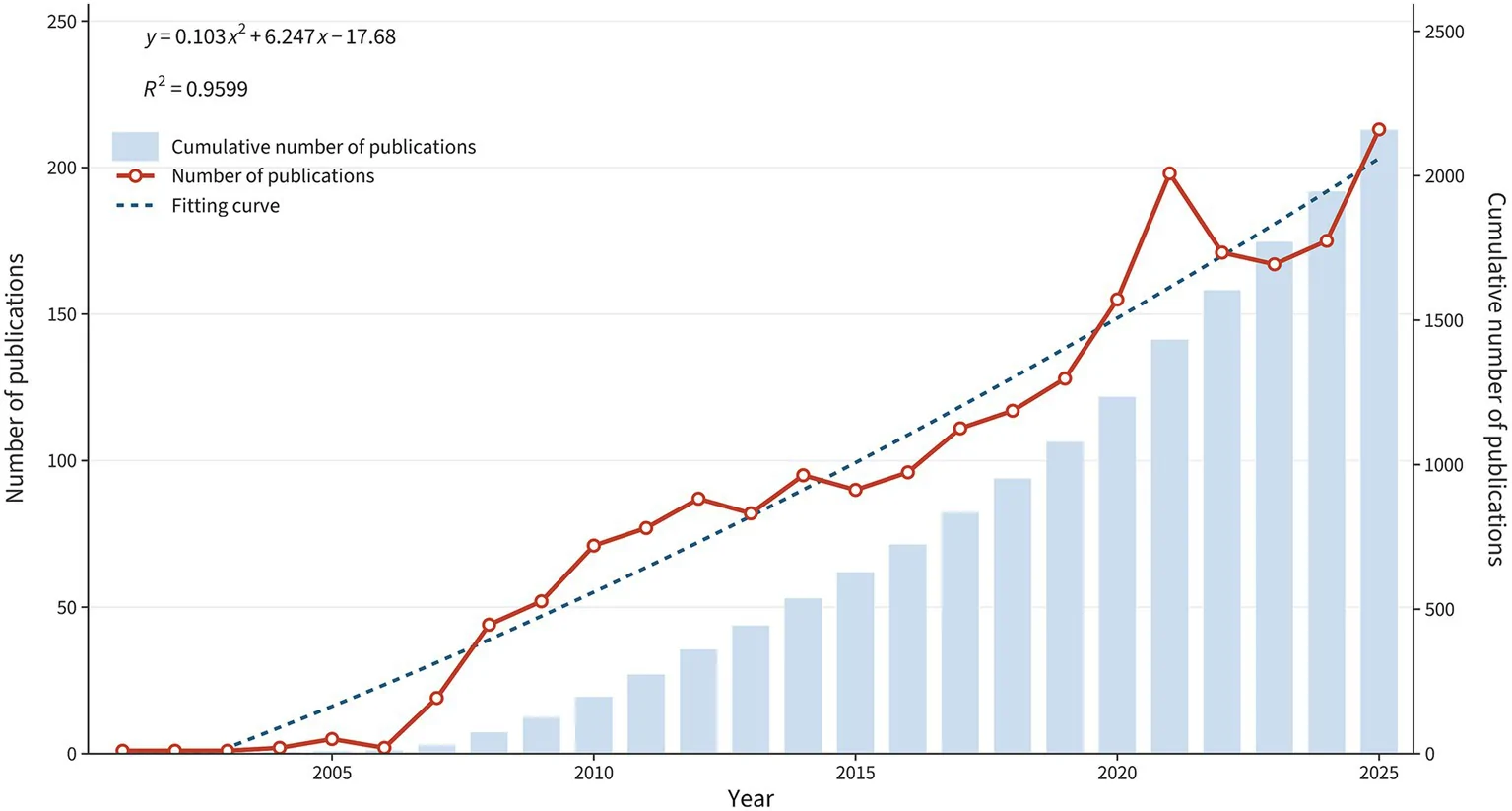
Annual scientific production, 2001–2025. Annual and cumulative publications with a second-order descriptive fit. The model summarizes observed growth and is not a forecast.

*Acta Neuropathologica* was the leading source with 118 publications, followed by *Acta Neuropathologica Communications* and the *Journal of Biological Chemistry* with 68 each. *Human Molecular Genetics* contributed 64 and the *International Journal of Molecular Sciences* 58. Bradford zone 1 contained 13 journals and 716 publications (33.15% of the corpus), combining neuropathology, molecular neuroscience, genetics, and biochemistry (Figure 3; Table 3).

**Figure 3:**
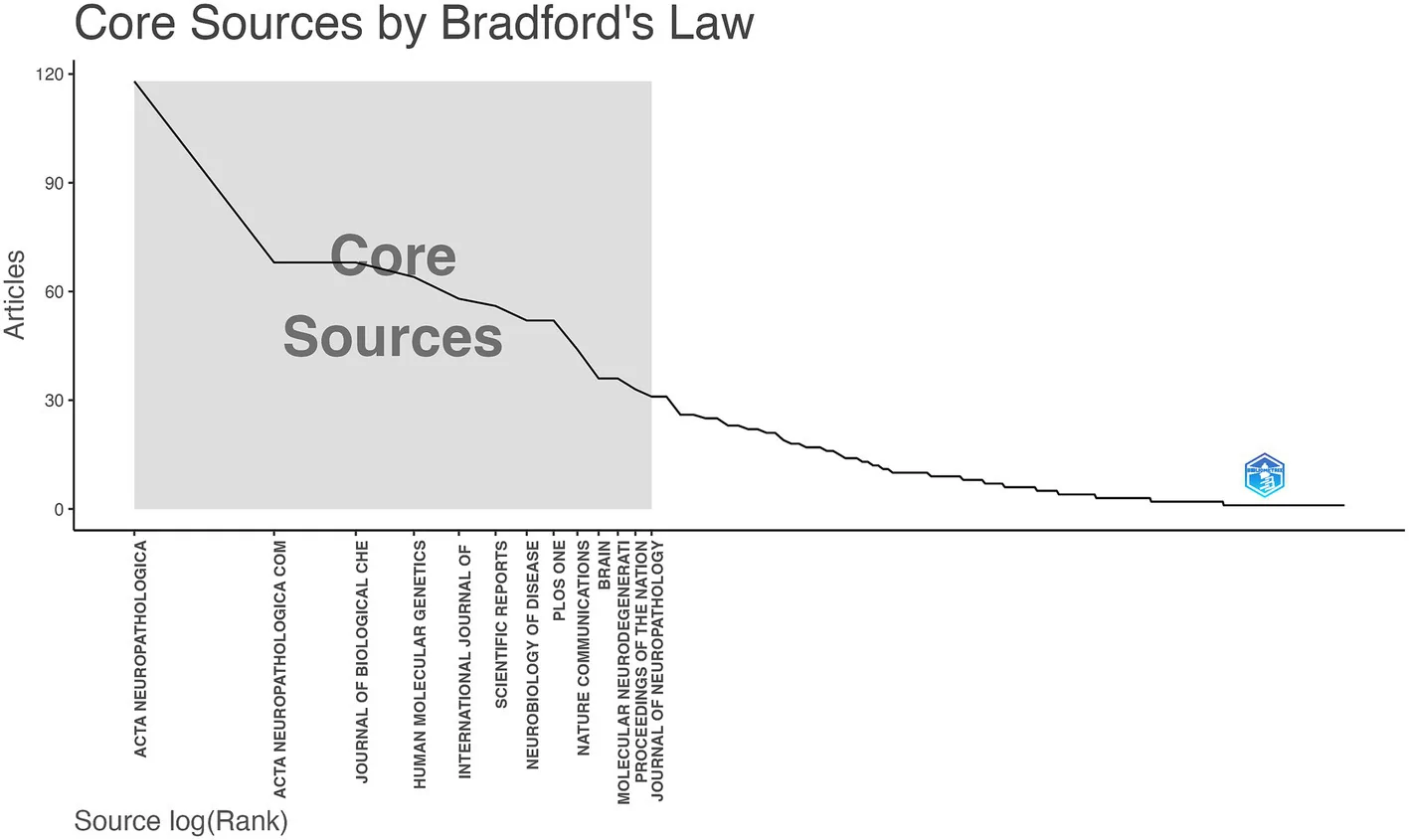
Leading journals and Bradford zones. Ranked publication output and the core, related, and peripheral source zones.

**Table 3:** Leading journals, authors, organizations, and countries.

Entity type	Rank	Entity	Documents	Additional indicator
Journal	1	Acta Neuropathologica	118	
Journal	2	Acta Neuropathologica Communications	68	
Journal	3	Journal of Biological Chemistry	68	
Journal	4	Human Molecular Genetics	64	
Journal	5	International Journal of Molecular Sciences	58	
Author	1	Dickson DW	78	
Author	2	Buratti E	76	
Author	3	Trojanowski JQ	66	
Author	4	Lee VMY	60	
Author	5	Petrucelli L	57	
Organization	1	University of California System	369	Database-standardized umbrella entity
Organization	2	Mayo Clinic	320	
Organization	3	University of Pennsylvania	281	
Organization	4	University of London	252	Database-standardized umbrella entity
Organization	5	University College London	141	
Country	1	USA	923	MCP 358 (38.8%)
Country	2	Japan	295	MCP 83 (28.1%)
Country	3	United Kingdom	254	MCP 197 (77.6%)
Country	4	Italy	214	MCP 110 (51.4%)
Country	5	China	208	MCP 83 (39.9%)

Documents were full-counted within entity type. Country MCP percentages represent the proportion of a country's publications with at least one foreign-country affiliation. Database-standardized umbrella organizations may overlap with constituent institutions.

### 3.3 Contributors and collaboration structure

Dickson DW was the most prolific author (78 publications), followed by Buratti E (76), Trojanowski JQ (66), Lee VMY (60), Petrucelli L (57), and Hasegawa M (55). The leading standardized organizational entities were the University of California System (369), Mayo Clinic (320), University of Pennsylvania (281), University of London (252), and University College London (141). Counts for umbrella systems and constituent institutions should not be combined.

The United States appeared in 923 publications, followed by Japan (295), the United Kingdom (254), Italy (214), China (208), Canada (174), Germany (153), and Australia (131). The United States had the greatest absolute number of internationally coauthored publications (358), whereas the MCP proportion was higher in the United Kingdom (77.6%) and Germany (70.6%). The strongest bilateral links were USA–United Kingdom (88 joint publications), China–USA (55), Germany–USA (49), Canada–USA (48), and Italy–USA (42). The 40-country VOSviewer network contained 264 links and showed a dense transatlantic core with additional East Asian and European clusters (Figure 4; Table 3).

**Figure 4:**
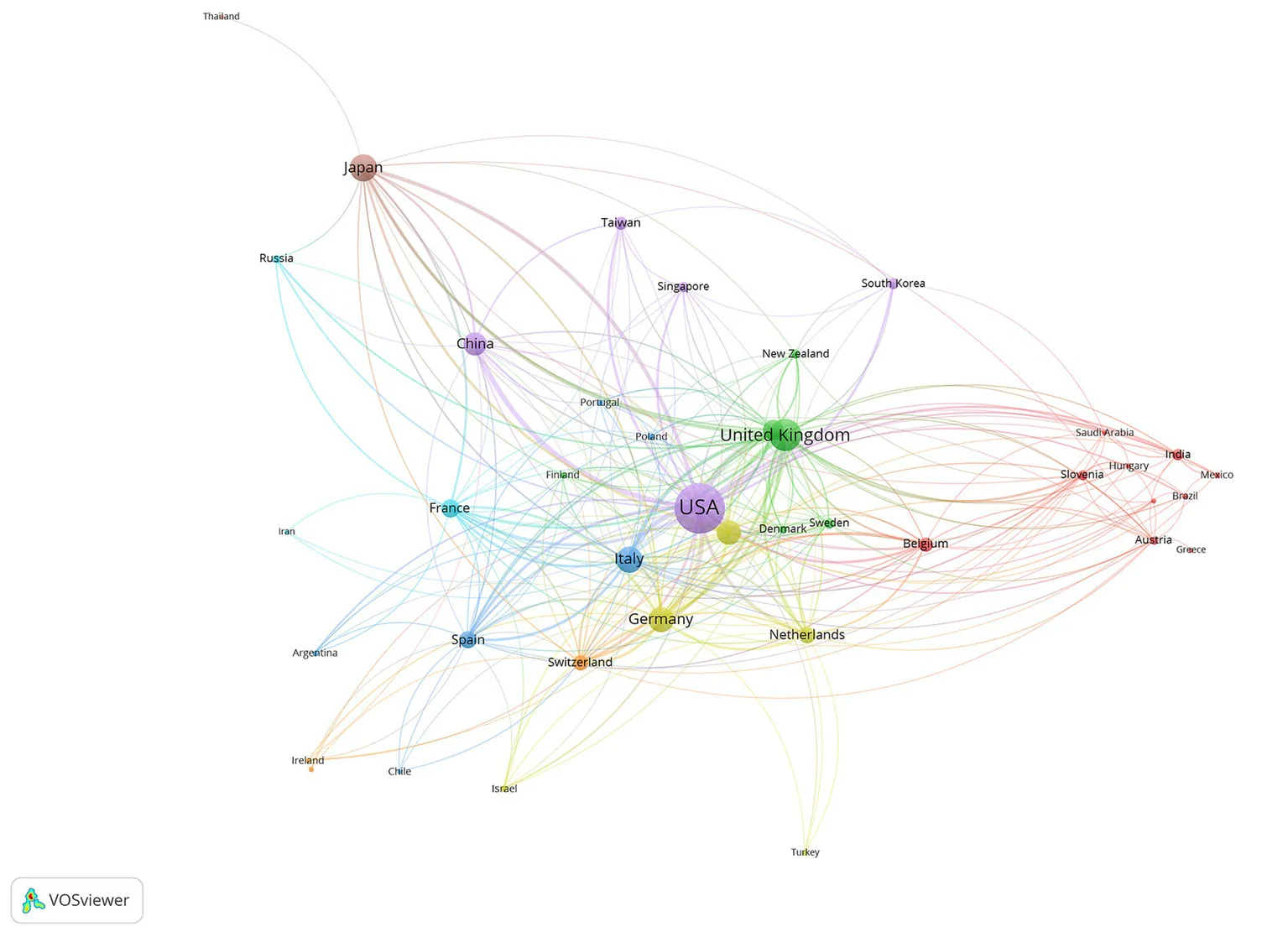
Country collaboration network. VOSviewer network visualization based on full-counted country co-authorship. Node size indicates country output, link thickness indicates collaboration strength, and colors identify collaboration clusters.

### 3.4 Foundational documents and the intellectual base

The most globally cited publication was Neumann et al.’s (2006) identification of ubiquitinated TDP-43 in FTLD and ALS (5,360 citations), followed by Arai et al.’s independent report (2,237), the discovery of pathogenic *TARDBP* variants by Sreedharan et al. (2,163), the hnRNPA2B1/hnRNPA1 multisystem proteinopathy study by Kim et al. (1,207), and the LATE consensus report (1,142). Polymenidou et al. (1,052) and Tollervey et al. (935) were the leading transcriptome-scale loss-of-function studies (Neumann et al., 2006; Arai et al., 2006; Sreedharan et al., 2008; Polymenidou et al., 2011; Tollervey et al., 2011; Nelson et al., 2019; Kim et al., 2013).

The primary VOSviewer co-citation map retained 60 core references linked by 1,770 co-citation relationships (Figure 5). Its largest and most central nodes recovered three connected foundations: neuropathological identification of TDP-43; genetic causation involving *TARDBP*, *FUS*, and other RBPs; and RNA-processing mechanisms. A complementary 80-node CiteSpace-compatible network had 221 edges, modularity *Q* = 0.4679, and weighted silhouette *S* = 1.000. The convergence of the two networks was more informative than direct comparison of their edge counts, which arise from different selection and construction rules.

**Figure 5:**
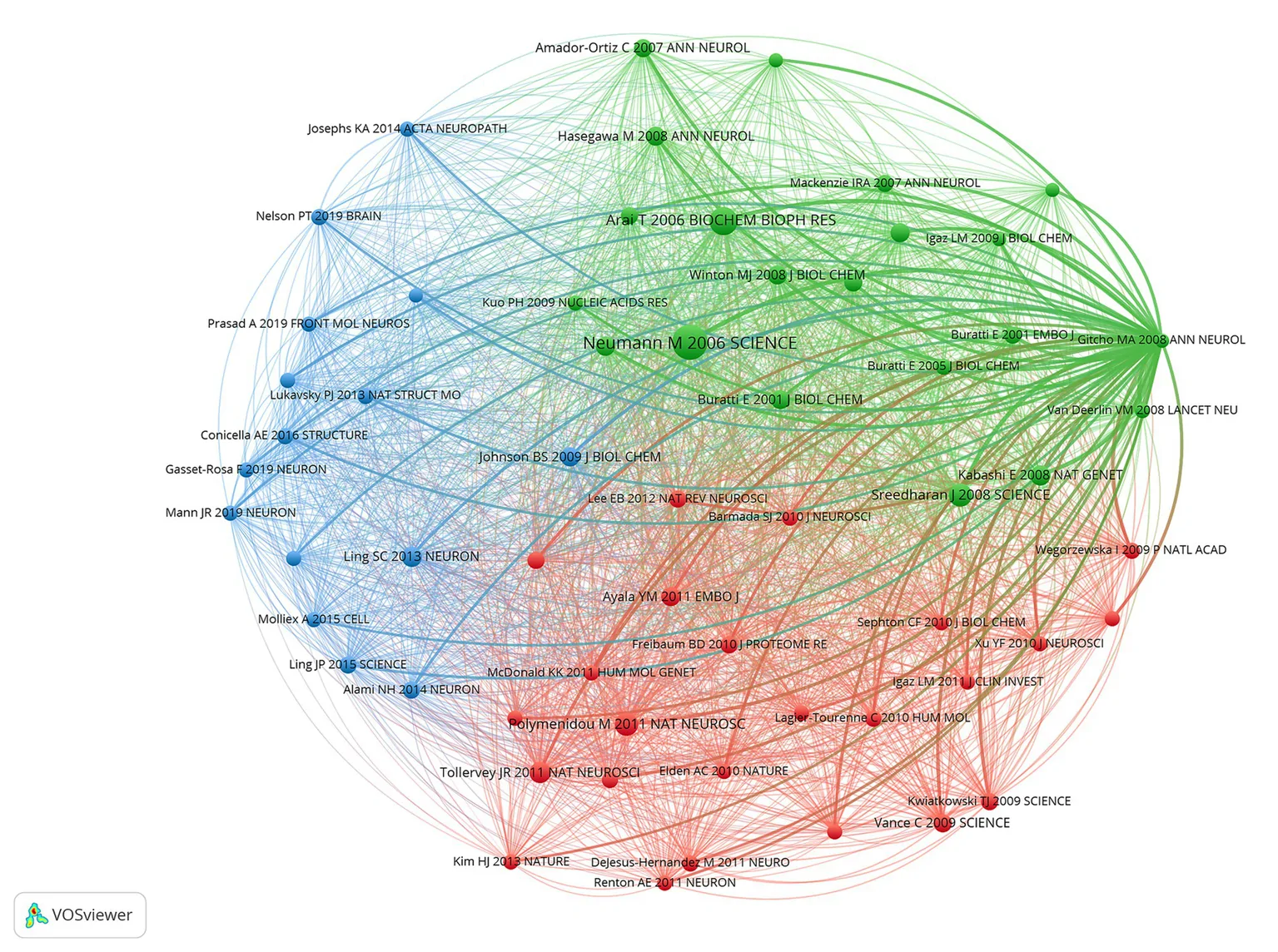
Co-cited reference network. VOSviewer visualization of the 60-reference core knowledge base. Node size indicates co-citation weight, link thickness indicates co-citation strength, and colors identify reference clusters.

### 3.5 Conceptual architecture

After thesaurus cleaning, the most frequent author keywords were TDP-43 (832 occurrences), amyotrophic lateral sclerosis (716), neurodegeneration (194), frontotemporal lobar degeneration (190), frontotemporal dementia (165), Alzheimer’s disease (134), RNA-binding proteins (119), stress granules (69), protein aggregation (67), tau (61), autophagy (50), FUS (50), and motor neuron disease (50).

The controlled title/abstract network contained 43 intact phrases and 682 links. It separated into three macro-communities (Figure 6). The first centered on TDP-43, ALS, FTLD/FTD, *TARDBP*, *C9orf72*, autophagy, mitochondrial dysfunction, apoptosis, microglia, astrocytes, and neuroinflammation. The second connected RNA-binding proteins, FUS/hnRNPs, RNA metabolism and splicing, nucleocytoplasmic and RNA transport, stress granules, liquid–liquid phase separation, prion-like domains, and proteostasis. The third comprised Alzheimer’s disease, tau, LATE, Parkinson’s disease, alpha-synuclein, amyloid beta, hippocampal sclerosis, and Huntington’s disease. The controlled network therefore separated a disease-dominant TDP-43 axis from an RBP/RNA-mechanism axis while placing late-life and non-ALS proteinopathies in a smaller cross-disease community.

**Figure 6:**
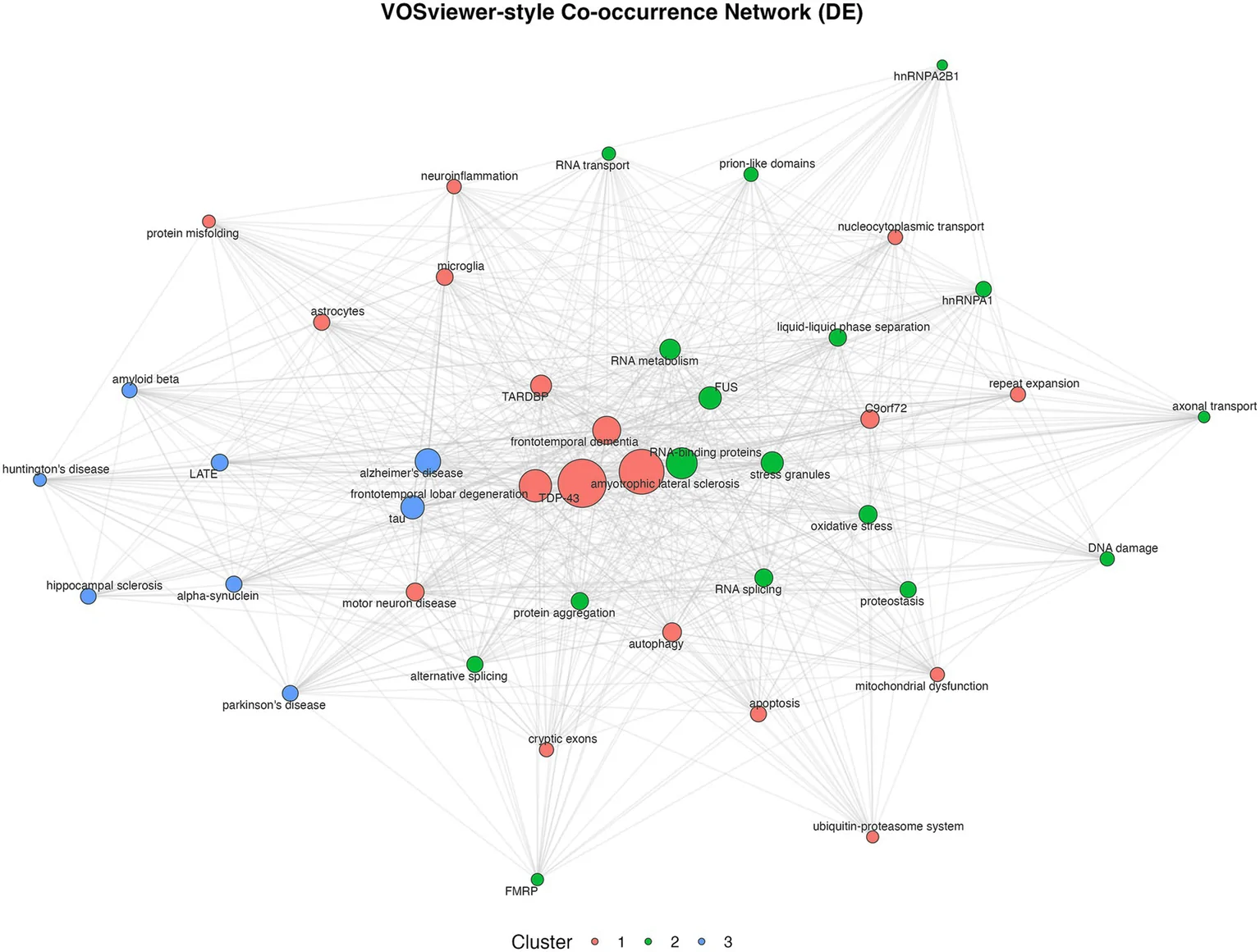
Phrase-preserving title/abstract co-occurrence network. Forty-three controlled disease, RBP, and mechanism phrases grouped into three VOS macro-communities. Multiword entities were matched before network construction and were not split into component words.

### 3.6 Temporal evolution and emerging fronts

The VOSviewer overlay placed established neuropathological and motor-neuron concepts earlier than cryptic exons, LATE, liquid–liquid phase separation, and RNA transport (Figure 7). Liquid–liquid phase separation had a mean publication year of 2021.86, and LATE had a mean year of 2022.66. In the density view, TDP-43 and amyotrophic lateral sclerosis formed the two brightest cores, followed by FTLD and RNA-binding proteins (Figure 8). Thus, recent concepts were visible at the network periphery, but they had not displaced the field’s longstanding concentration.

**Figure 7:**
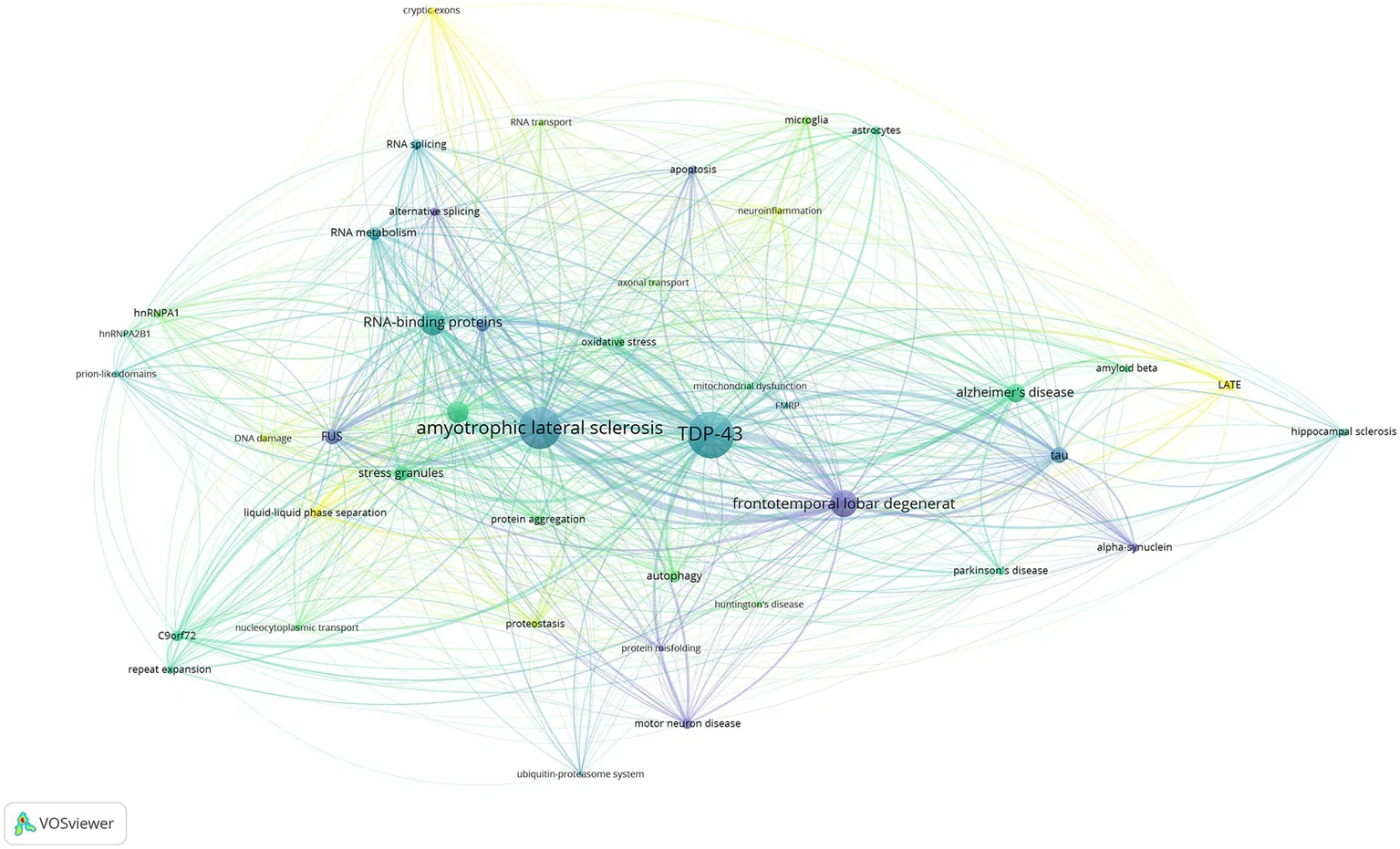
Controlled-concept temporal overlay. VOSviewer overlay of the same 43 phrases. Node color represents mean publication year, from earlier topics in blue–purple to more recent topics in yellow.

**Figure 8:**
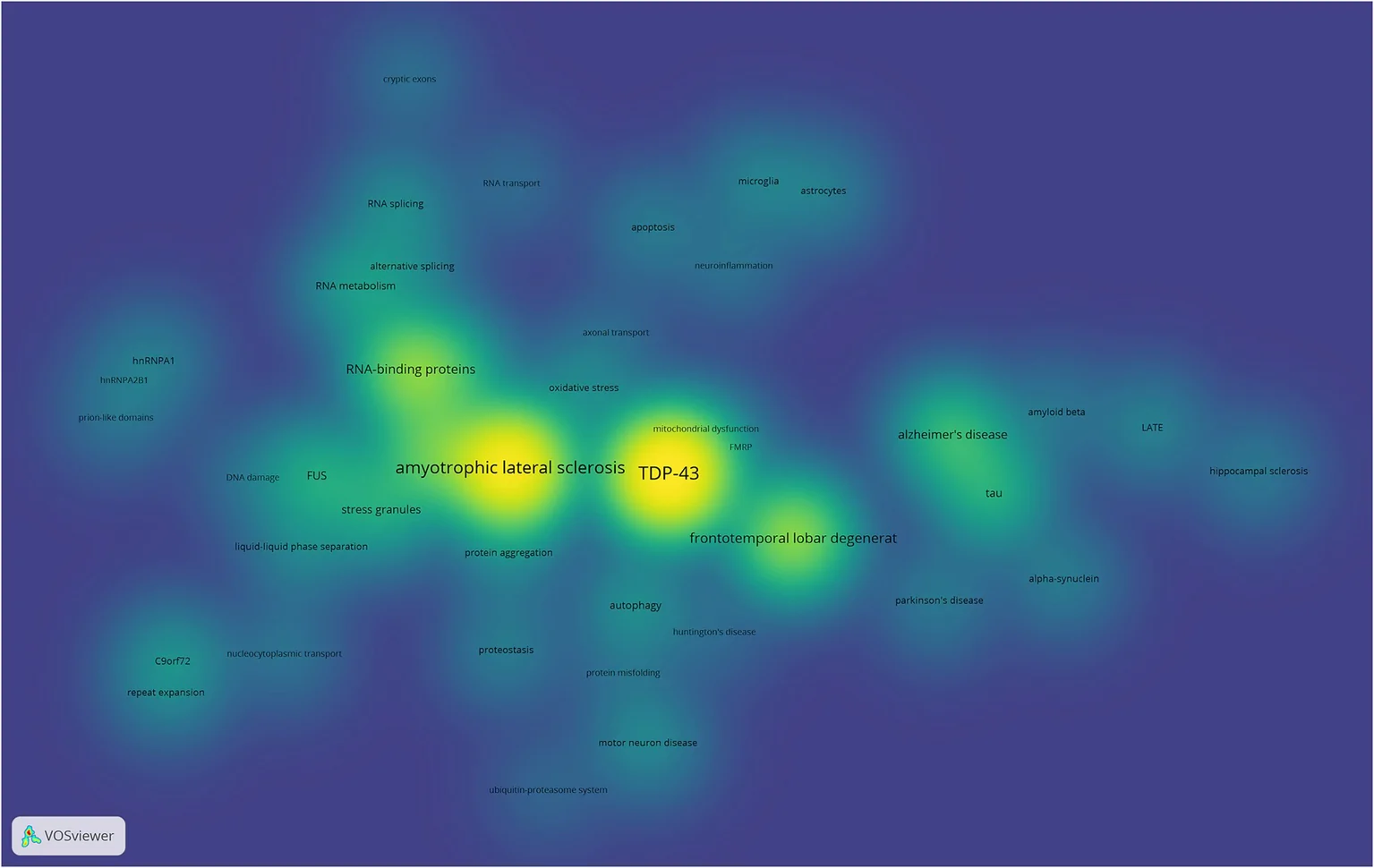
Controlled-concept density visualization. Brighter yellow regions indicate a higher weighted concentration of frequent and strongly connected concepts.

The thematic map positioned a broad mutation/disease/expression/aggregation complex as a central basic theme. The ALS/FTLD/RNA-processing complex showed higher internal development, whereas the Alzheimer disease/dementia/pathology complex had lower centrality and intermediate density (Figure 9). This configuration supports strong specialization around ALS/FTD and weaker integration of other neurodegenerative diseases.

**Figure 9:**
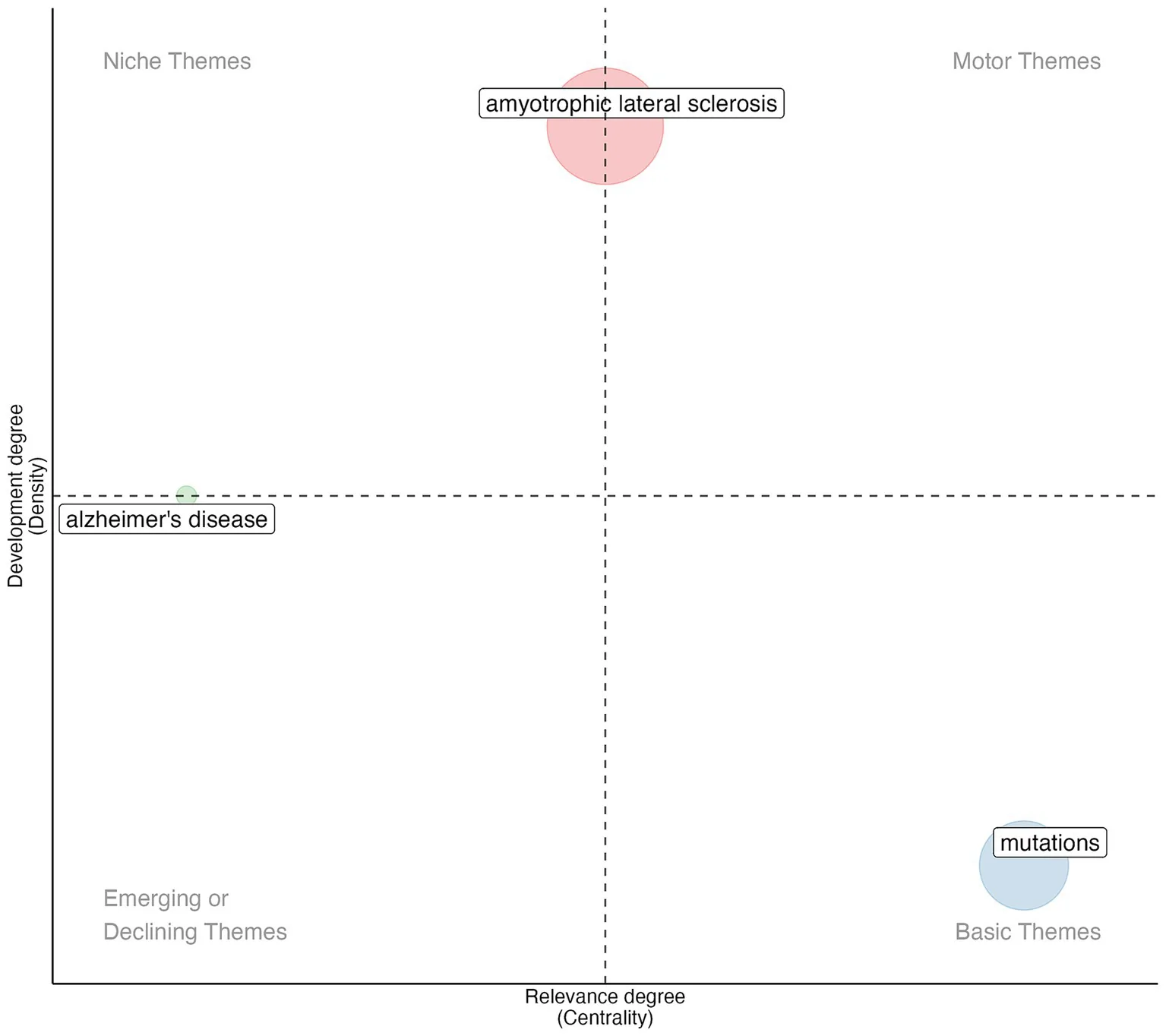
Thematic map. Keyword communities positioned by network centrality and density. Labels summarize source terms and should be interpreted as bibliometric themes rather than biological pathways.

The controlled burst analysis traced an early progression from alternative splicing (strength 2.71; 2001–2007), RNA-binding proteins (3.14; 2002–2005), and tau (8.61; 2002–2009) to motor neuron disease (14.27; 2005–2012), FTLD (41.80; 2006–2014), and FUS (9.49; 2010–2012). More recent bursts included nucleocytoplasmic transport (6.41; 2018–2022), liquid–liquid phase separation (5.44; 2021–2022), Parkinson’s disease (3.36; 2021–2023), protein aggregation (3.07; 2022–2023), LATE (12.56; 2022–2025), cryptic exons (8.02; 2022–2025), Huntington’s disease (3.44; 2023–2024), and RNA transport (2.82; 2024–2025). A separate author-keyword frequency analysis produced longer intervals for several high-frequency topics; this was treated as a field- and algorithm-sensitivity result rather than combined with the controlled burst estimates.

## 4 Discussion

### 4.1 Principal findings

This study maps a rapidly growing literature with pronounced intellectual concentration. Three bibliometric observations are central. First, TDP-43 and ALS/FTD occupy the most prominent positions across frequency, co-citation, and density analyses of the retrieved corpus. Second, the temporal maps show increasing attention to cryptic RNA processing, nuclear transport, RNA localization, and condensate biology. Third, LATE, Alzheimer disease, Huntington’s disease, and glial research are detectable but less integrated with the mature ALS/FTD literature. These are patterns of research activity and citation organization; mechanistic statements below summarize the cited experimental or clinicopathological literature and contextualize, rather than derive from, the bibliometric maps.

The mapped prominence should not be read as a ranking of biological importance. It is shaped by landmark discoveries, terminology, model availability, funding, community structure, database coverage, and the title-level query itself. Co-occurrence and co-citation identify topical and intellectual proximity but cannot establish biological interaction, directionality, therapeutic efficacy, or causality.

The study also differs from previous bibliometric work in scope and design. Earlier studies mapped ALS as a whole (Shi et al., 2022), FTD as a whole (Chen et al., 2024), or the most highly cited papers across several neurodegenerative diseases (Liao et al., 2021). Here, the cross-disease unit is RBP research itself. The added value is therefore not another disease-wide productivity ranking, but a phrase-preserving map of how RBP-centered mechanisms and disease communities connect within a clearly defined, title-anchored and PubMed-verified corpus.

### 4.2 TDP-43 path dependence and the ALS/FTD knowledge base

The co-citation structure paralleled the historical sequence described in mechanistic reviews: pathological identification, genetic validation, and functional dissection of RNA processing (Mackenzie et al., 2010; Lagier-Tourenne et al., 2010). Within the retrieved corpus, TDP-43 prominence is consistent with a discovery cascade linking pathology, causal variants, transcriptome-wide targets, model systems, and disease-spanning neuropathology. The cited experimental literature further showed that TDP-43 is intrinsically aggregation-prone (Johnson et al., 2009). Nevertheless, because TDP-43 was explicitly named in the title-level search, its observed bibliometric prominence cannot be separated from retrieval advantage in the present study and should not be generalized to the entire RBP literature.

However, this success creates path dependence. Concepts and diseases that enter through TDP-43 are more likely to connect to the established citation core, whereas RBPs outside the ALS/FTD axis may remain fragmented across disease-specific literatures. The genetic links of hnRNPA2B1/hnRNPA1 and ataxin-2 to ALS-spectrum phenotypes show that this broader RBP space is biologically consequential (Kim et al., 2013; Elden et al., 2010), and ataxin-2 reduction extends survival in a TDP-43 mouse model, illustrating how a less central RBP can become a therapeutic modifier of the dominant pathway (Becker et al., 2017). Even within ALS/FTD, RNA granule transport and local translation received less network weight than pathology and aggregation despite experimental evidence that ALS-causing mutations impair axonal transport of TDP-43 mRNA granules (Alami et al., 2014). Future mapping and experimental work should therefore distinguish genuine mechanistic convergence from the visibility advantage of a mature research program.

### 4.3 From RNA missplicing to actionable cryptic-exon biology

Within the retrieved corpus, cryptic exons formed a recent bibliometric front. The mechanistic trajectory is supported separately by experimental studies: TDP-43 loss reduces STMN2 through premature polyadenylation and impairs axonal growth and repair (Klim et al., 2019; Melamed et al., 2019); TDP-43 loss and ALS-risk alleles promote cryptic-exon inclusion and depletion of UNC13A (Brown et al., 2022; Ma et al., 2022); and subsequent work defined a correctable STMN2 splice-polyadenylation mechanism and hnRNP regulation of UNC13A cryptic splicing (Baughn et al., 2023; Koike et al., 2023). Thus, the maps identify rising research attention, whereas the cited experiments provide the evidence for molecular function and therapeutic tractability.

The cross-disease significance of this mechanism is still being defined. Cryptic STMN2 and UNC13A transcripts have been detected in TDP-43-associated Alzheimer disease (Agra Almeida Quadros et al., 2024), and large-scale RNA-sequencing approaches continue to identify candidate cryptic events (Sinha et al., 2025). Yet individual cryptic transcripts differ in abundance, tissue specificity, nonsense-mediated decay, and relationship to functional impairment. The field should therefore avoid treating any single cryptic exon as a universal surrogate for TDP-43 biology. Priority should be given to biomarkers that demonstrate analytical stability, regional and cell-type validity, and response to restoration of nuclear TDP-43 function.

### 4.4 Condensates, stress granules, and proteostasis

The bibliometric phase-separation cluster was recent and co-occurred with stress granules, prion-like domains, protein aggregation, autophagy, and nuclear transport. Its mechanistic context comes from experimental studies showing that low-complexity domains can drive stress-granule assembly and fibrillization, disease mutations can accelerate the liquid-to-solid transition of FUS, and ALS-associated changes can perturb TDP-43 phase behavior (Molliex et al., 2015; Patel et al., 2015; Conicella et al., 2016). TIA1 mutations connect altered phase separation and stress-granule dynamics to ALS/FTD genetics (Mackenzie et al., 2017), while RNA binding can oppose neurotoxic TDP-43 phase transitions (Mann et al., 2019). These studies, not the co-occurrence map alone, support the inference that condensate effects depend on molecular context.

This distinction is important because stress granules and pathological inclusions are related but not interchangeable. Cytoplasmic TDP-43 de-mixing can inhibit nuclear import and cause cell death independently of canonical stress granules (Gasset-Rosa et al., 2019). Conversely, cellular condensates can be adaptive responses to stress (Wolozin and Ivanov, 2019; Alberti and Hyman, 2021). Recent work on nuclear TDP-43 condensation and stress-granule-internal demixing suggests that pathogenicity may arise from altered material states or spatial transitions within condensates rather than from condensation alone (Vassallu and Igaz, 2024; Yan et al., 2025). The short controlled burst for liquid–liquid phase separation (2021–2022) should therefore be interpreted as a period of rapid vocabulary and concept adoption, not evidence that interest ended in 2022.

For therapeutic studies, “reducing phase separation” is too nonspecific an objective. Interventions should demonstrate which assembly state is modified, whether normal RNA processing is preserved, and whether target engagement improves neuronal function without disrupting adaptive granules.

### 4.5 Nuclear transport and neuroimmune coupling

Nucleocytoplasmic-transport terms bibliometrically bridged the RNA-processing and proteostasis communities. Experimental studies provide the mechanistic context: TDP-43 pathology can damage nuclear pore complexes (Chou et al., 2018), nuclear CHMP7 accumulation can initiate pore injury and subsequent TDP-43 dysfunction (Coyne et al., 2021), and C9orf72 repeat expansion can compromise nucleocytoplasmic transport (Freibaum et al., 2015; Zhang et al., 2015). ALS motor neurons also show mRNA redistribution alongside RBP mislocalization, with partial restoration after valosin-containing protein ATPase inhibition (Ziff et al., 2023). Collectively, the cited studies are consistent with bidirectional coupling, but neither they nor the bibliometric bridge establish one initiating event across genotypes.

The glial and neuroinflammatory subtheme was smaller but mechanistically diverse. TDP-43 can trigger mitochondrial DNA release and cGAS–STING activation (Yu et al., 2020); TREM2 can mediate microglial protection against TDP-43-associated degeneration (Xie et al., 2022); and progranulin haploinsufficiency can promote lysosomal abnormalities and cytoplasmic TDP-43 aggregation in human microglia (Sung et al., 2024). Recent astrocyte work connects neuroimmune signaling to nucleocytoplasmic disruption and stress-granule formation (Zhou et al., 2025). These studies caution against describing neuroinflammation as a uniformly downstream consequence. Depending on cell state and pathway, glial responses may be protective, maladaptive, or both over time. Bibliometric clustering can identify this convergence, but resolving directionality requires cell-type-specific and longitudinal experiments.

### 4.6 Expansion beyond ALS/FTD and implications for brain aging

The LATE/Alzheimer/tau community is directly relevant to aging neuroscience. TDP-43 pathology occurs in a substantial subset of cognitively normal older adults and becomes more prevalent with advancing age (Nascimento et al., 2018). LATE neuropathological change is concentrated in later life and commonly coexists with Alzheimer-type changes and hippocampal sclerosis, complicating attribution of cognitive decline to a single proteinopathy (Nelson et al., 2019). Selected cryptic exons also accumulate in Alzheimer hippocampus, including cases without conventionally classified TDP-43 proteinopathy (Torres et al., 2020), while emerging clinical criteria seek to identify a LATE syndrome during life (Wolk et al., 2025). The continuing LATE burst therefore indicates expanding research activity around a late-life clinicopathological and biomarker problem; it does not establish that ALS/FTD mechanisms operate unchanged in older adults with mixed pathology.

Cross-disease signals should nevertheless be interpreted cautiously. Huntington’s disease models and human tissue show G3BP1 granules and TDP-43 mislocalization (Sanchez et al., 2021), and recent work links disrupted TDP-43 activity to aberrant splicing and altered m6A regulation in Huntington’s disease (Nguyen et al., 2025). Interactions between alpha-synuclein and TDP-43 condensates provide a possible molecular bridge to synucleinopathies (Dhakal et al., 2023), but this evidence base is smaller and less mature than that for ALS/FTD. The network does not support assuming a single RBP mechanism across all neurodegenerative diseases. It instead identifies testable points of convergence that require validation in disease-relevant human tissue and models.

A practical strategy for testing cross-disease generalizability should proceed in stages. First, candidate processes highlighted by the ALS/FTD literature—including nuclear RBP loss of function, cryptic RNA processing, impaired nucleocytoplasmic or RNA transport, altered condensate and proteostasis states, and neuroimmune responses—should be measured with harmonized assays in neuropathologically defined cohorts spanning ALS/FTD, LATE, Alzheimer disease with and without TDP-43 pathology, other mixed proteinopathies, and age-matched controls. Matched brain regions should be analyzed with single-nucleus or spatial profiling, targeted splice-junction assays, quantitative proteomics, and digital pathology to distinguish vulnerable neurons from glial cell types. Second, signatures recurring across diseases should be tested for temporal order and causal relevance in patient-derived induced pluripotent stem-cell models, organoids, or neuron–glia co-cultures by pathway perturbation and rescue. Third, only mechanisms showing reproducible direction, cell-type specificity, association with neuropathological burden or clinical progression, and reversibility should advance to multicenter longitudinal biomarker studies or therapeutic testing. Harmonization of tissue processing, RNA quality, pathological classification, age, disease stage, ancestry, and co-pathology will be essential to distinguish a genuinely shared and actionable mechanism from parallel terminology or end-stage pathology.

### 4.7 Translational priorities and collaboration

The field’s translational opportunity lies in measuring RBP loss of function rather than relying solely on aggregate burden. Earlier biomarker studies found substantial heterogeneity in circulating and cerebrospinal-fluid TDP-43 measurements (Majumder et al., 2018). Cryptic RNA products, splice-junction signatures, and loss-of-function protein readouts may provide greater mechanistic specificity, but they require assay standardization, longitudinal validation, and comparison with neuropathological ground truth. Therapeutic development should connect antisense correction, restoration of nuclear RBP function, nuclear-pore rescue, proteostasis modulation, or neuroimmune intervention to a verified molecular target and neuronal outcome.

The collaboration network indicates that infrastructure is concentrated in a relatively small set of US, European, and Japanese centers. Strong bilateral links can accelerate replication, but global output alone does not guarantee diversity of biospecimens, ancestry, clinical phenotype, or disease stage. Multicenter consortia should prospectively harmonize tissue processing, RNA quality, pathological classification, and clinical metadata. Countries with high output but lower MCP proportions may be particularly well positioned to expand international validation.

### 4.8 Methodological implications, strengths, and limitations

Methodologically, this study shows why phrase preservation and semantic inspection are necessary in biomedical science mapping. An uncurated term pipeline represented “amyotrophic lateral sclerosis” as disconnected component words and produced generic nodes with little biological meaning. The controlled dictionary corrected this error and yielded a reproducible three-community structure. Similarly, invalid burst output caused by keyword-field concatenation was identified and rerun rather than reported. These corrections materially changed the interpretability of the conceptual results.

Strengths include a stored search strategy, explicit separation of database roles, preservation of raw and cleaned keyword fields, phrase-preserving concept mapping, age-normalized citation ranking, documented counting rules, complementary network methods, and rejected-output audits. PubMed verification provided a reproducible rule for defining the analysis corpus; it was not a quality assessment and did not justify pooling analytical fields from different databases. The interpretive population is therefore the WoS-derived literature successfully verified in PubMed, rather than all potentially relevant RBP publications.

Several limitations remain. First, the WoS–PubMed intersection may favor biomedical journals indexed in both databases. The 149 eligible-year WoS records not verified in PubMed were not available as a complete record-level analytical corpus in the archived revision materials, so their effects on contributor rankings, citation structures, conceptual networks, and temporal signals could not be quantified. We therefore do not claim robustness to retention of these records, and the conclusions are restricted to the 2,160-record verified corpus. Four interface records were also unavailable for evaluation. Second, restricting RBP terms to titles improves specificity but misses studies in which an RBP appears only in the abstract or full text. Naming TDP-43, FUS, and selected RBPs in that clause may increase their retrieval probability. Because an alternative-query analysis could not be reconstructed from the archived dataset, their prominence is reported only as a property of the query-defined corpus, without a claim of search-strategy robustness. Third, database indexing and citations are dynamic, and recent work remains disadvantaged by citation latency. Fourth, missing author keywords for one-third of records motivated, but did not externally validate, the title/abstract dictionary. Fifth, author and institution disambiguation remains imperfect. Sixth, clusters and bursts depend on fields, thresholds, normalization, and software, so node and edge counts should not be compared directly across methods. Finally, co-occurrence establishes topical proximity, not biological interaction or causality; one retracted record was retained for corpus reproducibility but was not used as mechanistic evidence.

## 5 Conclusion

The maps document a WoS-derived and PubMed-verified literature corpus organized around TDP-43 and ALS/FTD and increasingly connected with cryptic RNA processing, condensate biology, transport, proteostasis, neuroimmune research, and LATE. This concentration describes the retrieved research architecture and is not presented as an unbiased ranking of RBP biology or evidence of shared causality across diseases. The next phase should test candidate mechanisms in neuropathologically defined, age-stratified, and cell-type-resolved human cohorts, followed by causal perturbation and rescue in human disease models and multicenter longitudinal validation of reproducible biomarkers. Future bibliometric work using alternative query formulations and the complete WoS corpus will be needed to establish the stability and broader generalizability of the mapped architecture.

## Data Availability

The data analyzed in this study is subject to the following licenses/restrictions: the raw Web of Science records analyzed in this study are subject to database licensing restrictions and cannot be publicly redistributed. Derived aggregate tables, analysis code, and screening logs are available from the corresponding author upon reasonable request. Requests to access these datasets should be directed to Hongyan Li, lihongyan1019@126.com.

## References

[ref1] Agra Almeida Quadros A. R. Li Z. Wang X. Ndayambaje I. S. Aryal S. Ramesh N. . (2024). Cryptic splicing of stathmin-2 and UNC13A mRNAs is a pathological hallmark of TDP-43-associated Alzheimer's disease. Acta Neuropathol. 147:9. doi: 10.1007/s00401-023-02655-0, 38175301 PMC10766724

[ref2] Alami N. H. Smith R. B. Carrasco M. A. Williams L. A. Winborn C. S. Han S. S. W. . (2014). Axonal transport of TDP-43 mRNA granules is impaired by ALS-causing mutations. Neuron 81, 536–543. doi: 10.1016/j.neuron.2013.12.018, 24507191 PMC3939050

[ref3] Alberti S. Hyman A. A. (2021). Biomolecular condensates at the nexus of cellular stress, protein aggregation disease and ageing. Nat. Rev. Mol. Cell Biol. 22, 196–213. doi: 10.1038/s41580-020-00326-6, 33510441

[ref4] Arai T. Hasegawa M. Akiyama H. Ikeda K. Nonaka T. Mori H. . (2006). TDP-43 is a component of ubiquitin-positive tau-negative inclusions in frontotemporal lobar degeneration and amyotrophic lateral sclerosis. Biochem. Biophys. Res. Commun. 351, 602–611. doi: 10.1016/j.bbrc.2006.10.093 17084815

[ref5] Aria M. Cuccurullo C. (2017). Bibliometrix: an R-tool for comprehensive science mapping analysis. J. Informetr. 11, 959–975. doi: 10.1016/j.joi.2017.08.007

[ref6] Baughn M. W. Melamed Z. López-Erauskin J. Beccari M. S. Ling K. Zuberi A. . (2023). Mechanism of STMN2 cryptic splice-polyadenylation and its correction for TDP-43 proteinopathies. Science 379, 1140–1149. doi: 10.1126/science.abq5622, 36927019 PMC10148063

[ref7] Becker L. A. Huang B. Bieri G. Ma R. Knowles D. A. Jafar-Nejad P. . (2017). Therapeutic reduction of ataxin-2 extends lifespan and reduces pathology in TDP-43 mice. Nature 544, 367–371. doi: 10.1038/nature22038, 28405022 PMC5642042

[ref8] Brown A. L. Wilkins O. G. Keuss M. J. Kargbo-Hill S. E. Zanovello M. Lee W. C. . (2022). TDP-43 loss and ALS-risk SNPs drive mis-splicing and depletion of UNC13A. Nature 603, 131–137. doi: 10.1038/s41586-022-04436-3, 35197628 PMC8891020

[ref9] Chen C. (2006). CiteSpace II: detecting and visualizing emerging trends and transient patterns in scientific literature. J. Am. Soc. Inf. Sci. Technol. 57, 359–377. doi: 10.1002/asi.20317

[ref10] Chen X. Chen Y. Ni B. Huang C. (2024). Research trends and hotspots for frontotemporal dementia from 2000 to 2022: a bibliometric analysis. Front. Neurol. 15:1399600. doi: 10.3389/fneur.2024.1399600, 39087008 PMC11288951

[ref11] Chou C. C. Zhang Y. Umoh M. E. Vaughan S. W. Lorenzini I. Liu F. . (2018). TDP-43 pathology disrupts nuclear pore complexes and nucleocytoplasmic transport in ALS/FTD. Nat. Neurosci. 21, 228–239. doi: 10.1038/s41593-017-0047-3, 29311743 PMC5800968

[ref12] Conicella A. E. Zerze G. H. Mittal J. Fawzi N. L. (2016). ALS mutations disrupt phase separation mediated by α-helical structure in the TDP-43 low-complexity C-terminal domain. Structure 24, 1537–1549. doi: 10.1016/j.str.2016.07.007, 27545621 PMC5014597

[ref13] Coyne A. N. Baskerville V. Zaepfel B. L. Dickson D. W. Rigo F. Bennett F. . (2021). Nuclear accumulation of CHMP7 initiates nuclear pore complex injury and subsequent TDP-43 dysfunction in sporadic and familial ALS. Sci. Transl. Med. 13:eabe1923. doi: 10.1126/scitranslmed.abe1923, 34321318 PMC9022198

[ref14] Dhakal S. Mondal M. Mirzazadeh A. Banerjee S. Ghosh A. Rangachari V. (2023). α-Synuclein emulsifies TDP-43 prion-like domain-RNA liquid droplets to promote heterotypic amyloid fibrils. Commun. Biol. 6:1227. doi: 10.1038/s42003-023-05608-1, 38052886 PMC10697960

[ref15] Donthu N. Kumar S. Mukherjee D. Pandey N. Lim W. M. (2021). How to conduct a bibliometric analysis: an overview and guidelines. J. Bus. Res. 133, 285–296. doi: 10.1016/j.jbusres.2021.04.070

[ref16] Elden A. C. Kim H. J. Hart M. P. Chen-Plotkin A. S. Johnson B. S. Fang X. . (2010). Ataxin-2 intermediate-length polyglutamine expansions are associated with increased risk for ALS. Nature 466, 1069–1075. doi: 10.1038/nature09320, 20740007 PMC2965417

[ref17] Freibaum B. D. Lu Y. Lopez-Gonzalez R. Kim N. C. Almeida S. Lee K. H. . (2015). GGGGCC repeat expansion in C9orf72 compromises nucleocytoplasmic transport. Nature 525, 129–133. doi: 10.1038/nature14974, 26308899 PMC4631399

[ref18] Gasset-Rosa F. Lu S. Yu H. Chen C. Melamed Z. Guo L. . (2019). Cytoplasmic TDP-43 de-mixing independent of stress granules drives inhibition of nuclear import, loss of nuclear TDP-43, and cell death. Neuron 102, 339–357.e7. doi: 10.1016/j.neuron.2019.02.038, 30853299 PMC6548321

[ref19] Geuens T. Bouhy D. Timmerman V. (2016). The hnRNP family: insights into their role in health and disease. Hum. Genet. 135, 851–867. doi: 10.1007/s00439-016-1683-5, 27215579 PMC4947485

[ref20] Hasegawa M. Arai T. Nonaka T. Kametani F. Yoshida M. Hashizume Y. . (2008). Phosphorylated TDP-43 in frontotemporal lobar degeneration and amyotrophic lateral sclerosis. Ann. Neurol. 64, 60–70. doi: 10.1002/ana.21425, 18546284 PMC2674108

[ref21] Johnson B. S. Snead D. Lee J. J. McCaffery J. M. Shorter J. Gitler A. D. (2009). TDP-43 is intrinsically aggregation-prone, and ALS-linked mutations accelerate aggregation and increase toxicity. J. Biol. Chem. 284, 20329–20339. doi: 10.1074/jbc.M109.010264, 19465477 PMC2740458

[ref22] Kim H. J. Kim N. C. Wang Y. D. Scarborough E. A. Moore J. Diaz Z. . (2013). Mutations in prion-like domains in hnRNPA2B1 and hnRNPA1 cause multisystem proteinopathy and ALS. Nature 495, 467–473. doi: 10.1038/nature11922, 23455423 PMC3756911

[ref23] Klim J. R. Williams L. A. Limone F. Guerra San Juan I. Davis-Dusenbery B. N. Mordes D. A. . (2019). ALS-implicated protein TDP-43 sustains levels of STMN2, a mediator of motor neuron growth and repair. Nat. Neurosci. 22, 167–179. doi: 10.1038/s41593-018-0300-4, 30643292 PMC7153761

[ref24] Koike Y. Pickles S. Estades Ayuso V. Jansen-West K. Qi Y. A. Li Z. . (2023). TDP-43 and other hnRNPs regulate cryptic exon inclusion of a key ALS/FTD risk gene, UNC13A. PLoS Biol. 21:e3002028. doi: 10.1371/journal.pbio.3002028, 36930682 PMC10057836

[ref25] Lagier-Tourenne C. Polymenidou M. Cleveland D. W. (2010). TDP-43 and FUS/TLS: emerging roles in RNA processing and neurodegeneration. Hum. Mol. Genet. 19, R46–R64. doi: 10.1093/hmg/ddq137, 20400460 PMC3167692

[ref26] Liao Z. Wei W. Yang M. Kuang X. Shi J. (2021). Academic publication of neurodegenerative diseases from a bibliographic perspective: a comparative scientometric analysis. Front. Aging Neurosci. 13:722944. doi: 10.3389/fnagi.2021.722944, 34803653 PMC8601281

[ref27] Ling J. P. Pletnikova O. Troncoso J. C. Wong P. C. (2015). TDP-43 repression of nonconserved cryptic exons is compromised in ALS-FTD. Science 349, 650–655. doi: 10.1126/science.aab0983, 26250685 PMC4825810

[ref28] Ma X. R. Prudencio M. Koike Y. Vatsavayai S. C. Kim G. Harbinski F. . (2022). TDP-43 represses cryptic exon inclusion in the FTD-ALS gene UNC13A. Nature 603, 124–130. doi: 10.1038/s41586-022-04424-7, 35197626 PMC8891019

[ref29] Mackenzie I. R. Nicholson A. M. Sarkar M. Messing J. Purice M. D. Pottier C. . (2017). TIA1 mutations in amyotrophic lateral sclerosis and frontotemporal dementia promote phase separation and alter stress granule dynamics. Neuron 95, 808–816.e9. doi: 10.1016/j.neuron.2017.07.025, 28817800 PMC5576574

[ref30] Mackenzie I. R. Rademakers R. Neumann M. (2010). TDP-43 and FUS in amyotrophic lateral sclerosis and frontotemporal dementia. Lancet Neurol. 9, 995–1007. doi: 10.1016/S1474-4422(10)70195-2, 20864052

[ref31] Majumder V. Gregory J. M. Barria M. A. Green A. Pal S. (2018). TDP-43 as a potential biomarker for amyotrophic lateral sclerosis: a systematic review and meta-analysis. BMC Neurol. 18:90. doi: 10.1186/s12883-018-1091-7, 29954341 PMC6027783

[ref32] Mann J. R. Gleixner A. M. Mauna J. C. Gomes E. DeChellis-Marks M. R. Needham P. G. . (2019). RNA binding antagonizes neurotoxic phase transitions of TDP-43. Neuron 102, 321–338.e8. doi: 10.1016/j.neuron.2019.01.048, 30826182 PMC6472983

[ref33] Melamed Z. López-Erauskin J. Baughn M. W. Zhang O. Drenner K. Sun Y. . (2019). Premature polyadenylation-mediated loss of stathmin-2 is a hallmark of TDP-43-dependent neurodegeneration. Nat. Neurosci. 22, 180–190. doi: 10.1038/s41593-018-0293-z, 30643298 PMC6348009

[ref34] Molliex A. Temirov J. Lee J. Coughlin M. Kanagaraj A. P. Kim H. J. . (2015). Phase separation by low complexity domains promotes stress granule assembly and drives pathological fibrillization. Cell 163, 123–133. doi: 10.1016/j.cell.2015.09.015, 26406374 PMC5149108

[ref35] Nascimento C. Di Lorenzo Alho A. T. Bazan Conceição Amaral C. Leite R. E. P. Nitrini R. Jacob-Filho W. . (2018). Prevalence of TDP-43 proteinopathy in cognitively normal older adults: systematic review and meta-analysis. Neuropathol. Appl. Neurobiol. 44, 286–297. doi: 10.1111/nan.12430, 28793370 PMC5902737

[ref36] Nelson P. T. Dickson D. W. Trojanowski J. Q. Jack C. R. Boyle P. A. Arfanakis K. . (2019). Limbic-predominant age-related TDP-43 encephalopathy (LATE): consensus working group report. Brain 142, 1503–1527. doi: 10.1093/brain/awz099, 31039256 PMC6536849

[ref37] Neumann M. Sampathu D. M. Kwong L. K. Truax A. C. Micsenyi M. C. Chou T. T. . (2006). Ubiquitinated TDP-43 in frontotemporal lobar degeneration and amyotrophic lateral sclerosis. Science 314, 130–133. doi: 10.1126/science.1134108, 17023659

[ref38] Nguyen T. B. Miramontes R. Chillon-Marinas C. Maimon R. Vazquez-Sanchez S. Lau A. L. . (2025). Aberrant splicing in Huntington's disease accompanies disrupted TDP-43 activity and altered m6A RNA modification. Nat. Neurosci. 28, 280–292. doi: 10.1038/s41593-024-01850-w, 39762660 PMC11802453

[ref39] Nussbacher J. K. Batra R. Lagier-Tourenne C. Yeo G. W. (2015). RNA-binding proteins in neurodegeneration: Seq and you shall receive. Trends Neurosci. 38, 226–236. doi: 10.1016/j.tins.2015.02.003, 25765321 PMC4403644

[ref40] Page M. J. McKenzie J. E. Bossuyt P. M. Boutron I. Hoffmann T. C. Mulrow C. D. . (2021). The PRISMA 2020 statement: an updated guideline for reporting systematic reviews. BMJ 372:n71. doi: 10.1136/bmj.n71, 33782057 PMC8005924

[ref41] Patel A. Lee H. O. Jawerth L. Maharana S. Jahnel M. Hein M. Y. . (2015). A liquid-to-solid phase transition of the ALS protein FUS accelerated by disease mutation. Cell 162, 1066–1077. doi: 10.1016/j.cell.2015.07.047, 26317470

[ref42] Polymenidou M. Lagier-Tourenne C. Hutt K. R. Huelga S. C. Moran J. Liang T. Y. . (2011). Long pre-mRNA depletion and RNA missplicing contribute to neuronal vulnerability from loss of TDP-43. Nat. Neurosci. 14, 459–468. doi: 10.1038/nn.2779, 21358643 PMC3094729

[ref43] Sanchez I. I. Nguyen T. B. England W. E. Lim R. G. Vu A. Q. Miramontes R. . (2021). Huntington's disease mice and human brain tissue exhibit increased G3BP1 granules and TDP43 mislocalization. J. Clin. Invest. 131:e140723. doi: 10.1172/JCI140723, 33945510 PMC8203471

[ref44] Shi G. Zhou J. Huang K. Bi F. F. (2022). Trends in global amyotrophic lateral sclerosis research from 2000 to 2022: a bibliometric analysis. Front. Neurosci. 16:965230. doi: 10.3389/fnins.2022.965230, 36033620 PMC9399758

[ref45] Sinha I. R. Sandal P. S. Spence H. Burns G. D. Mallika A. P. Abbasinejad F. . (2025). Large-scale RNA-Seq mining reveals ciclopirox olamine induces TDP-43 cryptic exons. Nat. Commun. 16:6878. doi: 10.1038/s41467-025-62004-5, 40715064 PMC12297563

[ref46] Sreedharan J. Blair I. P. Tripathi V. B. Hu X. Vance C. Rogelj B. . (2008). TDP-43 mutations in familial and sporadic amyotrophic lateral sclerosis. Science 319, 1668–1672. doi: 10.1126/science.1154584, 18309045 PMC7116650

[ref47] Sung W. Noh M. Y. Nahm M. Kim Y. S. Ki C. S. Kim Y. E. . (2024). Progranulin haploinsufficiency mediates cytoplasmic TDP-43 aggregation with lysosomal abnormalities in human microglia. J. Neuroinflammation 21:47. doi: 10.1186/s12974-024-03039-1, 38347588 PMC10863104

[ref48] Taylor J. P. Brown R. H. Jr. Cleveland D. W. (2016). Decoding ALS: from genes to mechanism. Nature 539, 197–206. doi: 10.1038/nature20413, 27830784 PMC5585017

[ref49] Tollervey J. R. Curk T. Rogelj B. Briese M. Cereda M. Kayikci M. . (2011). Characterizing the RNA targets and position-dependent splicing regulation by TDP-43. Nat. Neurosci. 14, 452–458. doi: 10.1038/nn.2778, 21358640 PMC3108889

[ref50] Torres P. Andrés-Benito P. Fernàndez-Bernal A. Ricart M. Ayala V. Pamplona R. . (2020). Selected cryptic exons accumulate in hippocampal cell nuclei in Alzheimer's disease with and without associated TDP-43 proteinopathy. Brain 143:e20. doi: 10.1093/brain/awaa013, 32016361

[ref51] van Eck N. J. Waltman L. (2010). Software survey: VOSviewer, a computer program for bibliometric mapping. Scientometrics 84, 523–538. doi: 10.1007/s11192-009-0146-3, 20585380 PMC2883932

[ref52] Vance C. Rogelj B. Hortobágyi T. De Vos K. J. Nishimura A. L. Sreedharan J. . (2009). Mutations in FUS, an RNA processing protein, cause familial amyotrophic lateral sclerosis type 6. Science 323, 1208–1211. doi: 10.1126/science.1165942, 19251628 PMC4516382

[ref53] Vassallu F. Igaz L. M. (2024). TDP-43 nuclear condensation and neurodegenerative proteinopathies. Trends Neurosci. 47, 849–850. doi: 10.1016/j.tins.2024.09.003 39327159

[ref54] Wolk D. A. Nelson P. T. Apostolova L. Arfanakis K. Boyle P. A. Carlsson C. M. . (2025). Clinical criteria for limbic-predominant age-related TDP-43 encephalopathy. Alzheimers Dement. 21:e14202. doi: 10.1002/alz.14202, 39807681 PMC11772733

[ref55] Wolozin B. Ivanov P. (2019). Stress granules and neurodegeneration. Nat. Rev. Neurosci. 20, 649–666. doi: 10.1038/s41583-019-0222-5, 31582840 PMC6986315

[ref56] Xie M. Liu Y. U. Zhao S. Zhang L. Bosco D. B. Pang Y. P. . (2022). TREM2 interacts with TDP-43 and mediates microglial neuroprotection against TDP-43-related neurodegeneration. Nat. Neurosci. 25, 26–38. doi: 10.1038/s41593-021-00975-6, 34916658 PMC8741737

[ref57] Yan X. Kuster D. Mohanty P. Nijssen J. Pombo-García K. Garcia Morato J. . (2025). Intra-condensate demixing of TDP-43 inside stress granules generates pathological aggregates. Cell 188, 4123–4140.e18. doi: 10.1016/j.cell.2025.04.039, 40412392 PMC12303766

[ref58] Yu C. H. Davidson S. Harapas C. R. Hilton J. B. Mlodzianoski M. J. Laohamonthonkul P. . (2020). TDP-43 triggers mitochondrial DNA release via mPTP to activate cGAS/STING in ALS. Cell 183, 636–649.e18. doi: 10.1016/j.cell.2020.09.020, 33031745 PMC7599077

[ref59] Zhang K. Donnelly C. J. Haeusler A. R. Grima J. C. Machamer J. B. Steinwald P. . (2015). The C9orf72 repeat expansion disrupts nucleocytoplasmic transport. Nature 525, 56–61. doi: 10.1038/nature14973, 26308891 PMC4800742

[ref60] Zhou C. Hardin E. J. Zimmer T. S. Jackvony S. Barnett D. Khobrekar N. . (2025). Neuroimmune signaling mediates astrocytic nucleocytoplasmic disruptions and stress granule formation associated with TDP-43 pathology. Neurobiol. Dis. 211:106939. doi: 10.1016/j.nbd.2025.106939, 40339618 PMC12240875

[ref61] Ziff O. J. Harley J. Wang Y. Neeves J. Tyzack G. Ibrahim F. . (2023). Nucleocytoplasmic mRNA redistribution accompanies RNA binding protein mislocalization in ALS motor neurons and is restored by VCP ATPase inhibition. Neuron 111, 3011–3027.e7. doi: 10.1016/j.neuron.2023.06.019, 37480846

